# Self-rated Physical, Mental, Oral, and Cognitive Health in Older Korean Immigrants: The Role of Health Indicators and Sociocultural Factors

**DOI:** 10.1007/s10903-020-01087-2

**Published:** 2020-09-29

**Authors:** Yuri Jang, Eun Young Choi, Hyunwoo Yoon, Nan Sook Park, David A. Chiriboga, Miyong T. Kim

**Affiliations:** 1 Edward R. Roybal Institute on Aging, Suzanne Dworak-Peck School of Social Work, University of Southern California, 669 West 34^th^ Street, Los Angeles, CA 90089-0411 USA; 2 Leonard Davis School of Gerontology, University of Southern California, Los Angeles, USA; 3 School of Social Work, Portland State University, Portland, USA; 4 School of Social Work, University of South Florida, Florida, USA; 5 Department of Child and Family Studies, University of South Florida, Florida, USA; 6 School of Nursing, University of Texas at Austin, Texas, USA

**Keywords:** self-rated health, health assessment, social network, acculturation, Asian Americans

## Abstract

**Objectives:**

Guided by the models of health assessment and social determinants of health, we examined predictors of self-rated physical, mental, oral, and cognitive health of older Korean immigrants.

**Methods:**

Data came from the Study of Older Korean Americans (SOKA; *N* = 2,061, Mean age = 73.2). Multivariate regression models of self-ratings of health were tested with health indicators (both domain-specific and other health indicators including chronic disease, functional disability, problems with teeth or gums, and cognitive function) and sociocultural factors (acculturation, social network, and ethnic community social cohesion).

**Results:**

For self-rated physical, mental, and oral health, indicators specific to the targeted domain played a primary role, with those of other health domains playing a secondary role. Acculturation and social network were significant predictors of all four measures.

**Discussion:**

Findings highlight the importance of holistic health assessment that considers a wide range of health domains as well as sociocultural contexts.

In response to disparities in health and healthcare, increasing attention has been paid to older immigrants [1–3]. In 2016, about 14% of the U.S. population age 65 and older were foreign-born, and this figure is projected to reach 32% by 2060 [4]. Older immigrants of Asian origin in particular are expected to grow in number exponentially, yet this population remains understudied and underserved [5–7]. The cultural and linguistic challenges faced by these immigrants not only heighten their vulnerabilities in health and healthcare, but also make them hard to reach in research and services [7,8]. For example, more than half of older Asian Americans have limited English proficiency [9], a problem compounded by the fact that Asian Americans encompass more than two dozen ethnic groups and more than 300 languages [10]. These challenges make it imperative to attend to their ethnic/cultural/linguistic diversities to ensure accurate health assessment and service planning.

The present study focuses on the health of older Korean immigrants in the United Sates. With an estimated population of 1.8 million, Korean Americans represent the fifth largest Asian American subgroup [10]. Because most Koreans have arrived in the United States since the Immigration Act of 1965, the current generation of older Korean Americans is predominantly foreign-born and challenged by linguistic and cultural barriers [10]. Among languages spoken by older immigrants with limited English proficiency, Korean ranks fourth just after Spanish, Chinese, and Russian [9]. Korean American vulnerabilities in health have been reported in many studies [2,11]; however, attention has been limited to physical and mental health. Recognizing the multidimensional complexities of health [12,13], we expanded our assessment to include oral and cognitive health.

Self-rated *physical* health (SRPH), a single item questioning “How would you rate your health?” has become well-established as an important health measure. SRPH is documented to predict older individuals’ general health and well-being and even mortality [14,15]. With its predictive power being equal to or exceeding that of objective health measures, SRPH has become a standard part of national and international health surveys, and it serves as an important tool in health screenings and clinical trials [16–18].

Self-rated *mental* health (SRMH) has also gained its prominence, and studies demonstrate its close linkage to mental health symptoms, psychiatric diagnosis, and the use of mental health services [19–21]. The relevance of the single-item self-rating approach is also found in studies of *oral* health [22,23] and *cognitive* health [8,24]. However, there is a dearth of knowledge on the mechanisms through which these self-ratings operate.

Typically ranging from *excellent* to *poor*, the response to the single-item self-rating question is quick but reflects a complex process. Building on an earlier work on the cognitive flow of health [25], Jylhä [17] proposed a model of subjective health assessment that involves information processing, interpretation of meanings, and response selection. The model posits that individuals primarily base their evaluation of health on biological and health-relevant factors. Further, it recognizes the social determinants of health [13,26]. Particularly in the domain of physical health, our understanding has been enriched by the consideration of social determinants that encompass life circumstances, personal and social resources, and environmental factors [13,14]. In the present study, we applied the model to self-ratings of physical, mental, oral, and cognitive health.

In considering self-ratings of these health domains, it is important to keep in mind that more objective health indicators relevant to each specific domain have generally been the primary sources of personal assessment. For example, in Jylhä’s model [17], SRPH is primarily driven by objective factors specific to physical health such as medical diagnosis, functional status, and bodily sensations and symptoms. In addition to these domain-specific health indicators, other dimensions of health such as depressive symptoms and problems with teeth or gums may serve as contextual variables that shape subjective assessment of physical health. In the present study, the domain-specific indicators are chronic disease and functional disability for SRPH, mental distress for SRMH, problems with teeth or gums for self-rated oral health (SROH), and cognitive function for self-rated cognitive health (SRCH). These health indicators can serve as either primary or secondary sources in any given assessment of physical, mental, oral, or cognitive health. For example, SRPH would be influenced primarily by variables specific to physical health (e.g., chronic disease and functional disability); however, mental distress, problems with teeth or gums, and cognitive function might also be contributing factors.

Beyond health indicators, sociocultural factors are another set of critical variables that influence self-assessment of health. For this study, non-health-related variables such as acculturation, social network, and ethnic community social cohesion were anticipated to serve as critical determinants of health. Each of these variables represents individual-, interpersonal-, and community-level resources, and its selection was based on the literature on immigrants’ health and social capital. For example, acculturation is widely known as an important personal asset that enhances immigrants’ health and well-being [27]. Further, studies demonstrate the broad health benefit of being connected with family and friends [28,29] and of feeling cohesive in ethnic communities [30,31].

In summary, we examined (1) how SRPH, SRMH, SROH, and SRCH might be associated with one another, and (2) how each of them might be predicted by health indicators (both domain-specific health indicators and other health indicators) and sociocultural factors (acculturation, social network, and ethnic community social cohesion). We hypothesized that (1) the self-rated measures of physical, mental, oral, and cognitive health would be interrelated and that (2) the set of domain-specific health indicators, other health indicators, and sociocultural factors would make a unique contribution to explaining each measure.

## Methods

### Participants

Data were drawn from the Study of Older Korean Americans (SOKA), a multi-state survey of Korean immigrants age 60 and older. In an effort to increase the generalizability of findings, sites were selected from states with differing proportions of the entire Korean population: California (29.3%), New York (8.0%), Texas (5.2%), Hawaii (2.7%), and Florida (2.2%) [4]. In each state, a primary metropolitan statistical area with a representative proportion of Korean Americans was selected: Los Angeles, New York, Austin, Honolulu, and Tampa. Combined, these sites present a continuum of Korean population densities. The study employed non-probability, culturally and linguistically sensitive sampling strategies. Probability sampling was not possible due to the absence of a sampling base that reflects the characteristics of the older Korean population; a majority have limited English proficiency and are undercounted in the Census [2,10].

Study participants were recruited by a team of investigators who shared the language and culture of the target population. The project began with the compiling of a database of Korean-oriented resources, services, and amenities at each study location; this database not only facilitated the research team’s efforts for community engagement but also guided the selection of specific locations for data collection. In the development of these databases and in their use at each site, community advisors’ input was actively solicited. At each of the five geographic sites, surveys took place at multiple locations and events (e.g., churches, temples, grocery stores, small group meetings, and cultural events) from April 2017 to February 2018. The survey questionnaire was in Korean, developed through a back-translation and reconciliation method. The questionnaire was designed to be self-administered, but trained interviewers were onsite for anyone who needed assistance. Upon completion of the survey, each participant was assessed for cognitive function, using the Mini-Mental State Examination (MMSE), by trained research personnel. All participants were paid U.S. $20 each for participation. The project was approved by a university’s Institutional Review Board. All participants were informed of the study’s goals and signed an informed consent form. A total of 2,176 individuals participated in the survey. After removal of those who had more than 10% of data missing on variables used in the present analyses (*n* = 111) or whose cognitive status suggested severe impairment (MMSE score <10; *n* = 4), the final sample consisted of 2,061 participants.

### Measures

#### Self-rated measures of health.

Participants were asked to rate their overall status in four health domains: physical, mental, oral, and cognitive health. Each domain was rated using a 5-point scale: 1 (*poor*), 2 (*fair*), 3 (*good*), 4 (*very good*), or 5 (*excellent*).

#### Health indicators.

As indicators specific to physical health, chronic disease and functional disability were used. The total count for a checklist of 10 chronic diseases and conditions common in older populations (e.g., diabetes, cancer, arthritis, heart disease, high blood pressure) was used as a continuous format. Functional disability was assessed with a composite measure [32] that includes activities of daily living (ADL) and instrumental activities of daily living (IADL). The scale comprises items for 16 activities (e.g., walking, bathing, dressing, managing medication), and participants were asked to indicate how well they could perform each activity. Responses were coded as 0 (*without help*), 1 (*with some help*), or 2 (*unable to do*). Total scores could range from 0 (*no functional disability*) to 32 (*severe functional disability*). Internal consistency of the scale in the present sample was high (α = .89).

As the indicator specific to mental health, level of mental distress was measured by the Kessler Psychological Distress Scale-6 (K6) [33,34]. Participants were asked to report how often, over the past 30 days, they had experienced such symptoms as “so depressed that nothing could cheer you up,” “hopeless,” and “everything was an effort.” Each item was rated on a 5-point scale ranging from 0 (*none of the time*) to 4 (*all of the time*). Responses were summed to create a composite score, ranging from 0 to 24. The K6 has been translated into Korean, and its psychometric properties have been validated in samples of Koreans and Korean Americans [35]. Cronbach’s alpha for the present sample was .91.

For the indicator specific to oral health, participants were asked whether they had any problems with teeth or gums (0 = *no*, 1 = *yes*). Cognitive function was indicated by MMSE [36] with items on time and place orientation, registration, attention, recall, language, and visual construction. Responses for each item were scored as 1 (*correct*) or 0 (*incorrect*). Total scores could range from 0 to 30, with higher scores indicating better cognitive function. The psychometric properties of the Korean version of the MMSE have been validated [37]; internal consistency in the present sample was satisfactory (α = .73).

#### Sociocultural factors.

Acculturation, social network, and ethnic community social cohesion were considered. The level of acculturation was assessed with a 12-item inventory of acculturation [27], addressing English proficiency, media consumption, food consumption, social relationship, sense of belonging, and familiarity with culture and customs. Each response was coded from 0 (*not at all*) to 3 (*very well/very often/very much*). Total scores could range from 0 to 36, with higher scores indicating a greater level of acculturation to mainstream American culture. Internal consistency in the present sample was high (α = .91).

Social network was measured with the six items in Lubben’s Social Network Scale-6 (LSNS-6) [38,39]. These questions asked about the number of family or friends seen at least once a month, the number with whom respondents felt at ease to discuss private matters, and the number to whom they felt close. Responses were given on a 6-point scale ranging from 0 (*none*) to 5 (*nine or more*). Total scores could range from 0 to 30, with higher scores indicating a stronger social tie. The scale has been translated into Korean, and it has been validated for psychometric properties [40]. Internal consistency in the present sample was high (α = .88).

Ethnic community social cohesion was measured with a five-item scale adapted from previous studies on social capital in general populations [41]. Participants were asked to indicate their level of agreement on such statements as “People in my ethnic community are willing to help each other,” “People in my ethnic community generally get along with each other,” and “People in my ethnic community share the same values.” Responses were given on a 5-point scale ranging from 0 (*strongly disagree*) to 4 (*strongly agree*). Total scores could range from 0 to 20, with higher scores indicating a greater level of sense of cohesion. Internal consistency in the present sample was high (α = .92).

#### Demographic variables.

Background information included age (in years), gender (0 = *male*, 1 = *female*), marital status (0 = *not married*, 1 = *married*), and education (0 = ≤*high school graduation*; 1 = >*high school graduation*).

### Analytical Strategy

Descriptive statistics and bivariate correlations were performed to understand the overall characteristics of the sample and underlying associations among study variables. The self-rated measures of health were each used in their continuous format in order to explore increments in the variance explained by sequential sets of theory-driven predictors. Series of linear regression models were separately tested for each of the four self-ratings of health. For each model, the sets of predictors were entered in the following order: (1) demographic variables, (2) domain-specific health indicators, (3) other health indicators, and (4) sociocultural variables. This approach allowed assessment of how each set of predictors independently contributed to the variance of the self-rated measures of health. All analyses were performed using IBM SPSS Statistics 25.

## Results

### Descriptive Characteristics

Table 1 summarizes the overall characteristics of the sample. The mean age was 73.2 years (*SD* = 7.93), with a range from 60 to 100. Approximately 67% of the sample were women, and over half (60.8%) were married. About 40% had attained formal education beyond high school. The scores of chronic disease, functional disability, and mental distress averaged 1.57 (*SD* = 1.40), 1.67 (*SD* = 3.42), and 3.88 (*SD* = 4.05), respectively. Reflecting the nature of a community-dwelling volunteer sample, these scores were skewed to the healthy side. For multivariate analyses, the scores were log-transformed to approximate normal distributions. More than 20% reported a problem with teeth or gums, and MMSE scores averaged 26.7 (*SD* = 2.91).

With regard to sociocultural factors, the mean scores for acculturation, social network, and community cohesion were 12.2 (*SD* = 7.06), 15.5 (*SD* = 6.05), and 11.4 (*SD* = 4.11), respectively. Concerning self-rated health assessments, the average score for oral health was the lowest (*M* = 2.61, *SD* = 1.29), followed by physical health (*M* = 3.02, *SD* = 1.24), cognitive health (*M* = 3.15, *SD* = 1.13), and mental health (*M* = 3.54, *SD* = 1.13). The proportions of the sample reporting *fair* or *poor* status were 36.4% for SRPH, 21.3% for SRMH, 52.3% for SROH, and 33.2% for SRCH.

### Correlations among Study Variables

Table 2 presents the bivariate correlations among the study variables. Each self-rated measure was significantly associated with its corresponding domain-specific health indicators in the expected direction: SRPH with chronic disease (*r* = −.47, *p* < .001) and functional disability (*r* = −.37, *p* < .001), SRMH with mental distress (*r* = −.36, *p* < .001), SROH with problem with teeth or gums (*r* = −.41, *p* < .001), and SRCH with cognitive function (*r* = .25, *p* < .001). Other health indicators non-specific to a given health measure also showed significant correlations. For example, chronic disease and functional disability were significantly correlated with a poorer rating of SRMH (*r* = −.31, *p* < .001 and *r* = −.32, *p* < .001, respectively), SROH (*r* = −.23, *p* < .001 and *r* = −.18, *p* < .001, respectively), and SRCH (*r* = −.24, *p* < .001 and *r* = −.23, *p* < .001, respectively). It is noteworthy that the association of SRCH with mental distress was even greater (*r* = −.31, *p* < .001) than that with cognitive function (*r* = .25, *p* < .001). Positive ratings of health were associated with high levels of acculturation (*r*s = .35− .37, *p*s < .001) and large social networks (*r*s = .21− .24, *p*s < .001). Community cohesion, however, had either non- significant or marginally significant correlations with the measures of self-rating. It is also worth noting that the self-rated measures of health were positively associated with one another. The strongest relationship was found between SRMH and SRCH (*r* = .67, *p* < .001). Similarly, the correlation coefficient between SRPH and SRMH exceeded .60. Because each measure was used as a separate outcome variable, there were no concerns about collinearity.

### Regression Models of Self-rated Measures of Health

Table 3 shows the results from the multivariate linear regression model of each self-rated health outcome. In the initial model of SRPH, the set of demographic variables explained 13% of the variance, and all variables were significant. Positive ratings of physical health were related to those who were younger, male, married, and with higher levels of education. In the subsequent entry of domain-specific indicators, both chronic disease and functional disability were associated with lower SRPH scores, increasing the explained variance by 21%. The addition of other health indicators contributed an additional 3% to the explained variance, with mental distress and problems with teeth or gums being significant predictors. In the final model with sociocultural factors, an additional 2% of the variance was explained. Positive SRPH was associated with higher levels of acculturation and larger social networks. The total variance explained by the estimated model was 39%.

A similar pattern was observed in the regression models of the other three outcome measures. Each entry of demographic variables, domain-specific health indicators, other health indicators, and sociocultural factors made unique contributions to the predictive models. In the final model of SRMH, advanced age, lower education, higher levels of mental distress, greater numbers of chronic diseases, greater level of functional disability, having problems with teeth or gums, poorer cognitive function, lower acculturation, and smaller social networks were associated with more adverse ratings of SRMH. The full model accounted for 33% of the variance.

In the final model of SROH, negative ratings were observed among those with male gender, lower education, and problems with teeth or gums. Among other health indicators, chronic disease and mental distress were predictive of negative ratings of SROH. All of the sociocultural factors reached statistical significance; those with greater levels of acculturation, social network, and community cohesion had more favorable SROH. The total variance explained by the model was 29%.

Finally, in the model of SRCH, higher educational attainment was associated with positive ratings. In addition to the MMSE scores, all of the other health indicators were significant predictors of SRCH. Mental distress had a substantial negative impact on SROH, but higher levels of acculturation and social connectedness promoted positive ratings of SROH. The total variance explained by the model was 28%.

## Discussion

In the present study, we examined factors associated with self-ratings of physical, mental, oral, and cognitive health in a sample of older Korean Americans. Based on the health assessment model posed by Jylhä [17] and the notion of social determinants of health [13,26], we explored the role of health indicators—both domain-specific and other health indicators— and sociocultural factors. Although specific score comparisons of self-rated health measures between the present sample and others in the literature are not feasible due to coding differences across studies, more than one third of the present sample (36.4%) reported fair/poor SRPH; one fifth (21.3%), fair/poor SRMH; and over half (52.3%), fair/poor SROH. These rates are notably higher than those observed in age-comparable samples of non-Hispanic Whites [42,43]. The rate of fair/poor SRCH (33.2%) was also slightly higher than the 27.1% observed in a national sample of U.S. older adults [24]. Overall, the findings are in line with previous studies reporting the health vulnerabilities of older Korean Americans, particularly in the areas of physical, mental, and oral health [2,11].

As anticipated, all measures of health ratings were positively correlated, indicating intersectionality among different dimensions of health. Poor ratings of one dimension of health were likely to be linked to poor ratings of other dimensions of health. The close linkage between SRPH and SRMH (*r* = .62, *p* < .001) was anticipated because many studies have shown a close link between physical and mental health [44]. The present sample also demonstrated a significant correlation between SRMH and SRCH (*r* = .67, *p* < .001), and this finding is in line with literature demonstrating close connections between the symptoms of cognitive impairment (e.g., lack of ability to pay or sustain attention and problems with memory) and those of mood disorders [45].

Unique to the present study, health indicators were divided into domain-specific and other indicators, and their independent contributions to the predictive model of self-ratings of health were confirmed. The domain-specific health indicators for each measure served as a fundamental base of health assessment, contributing to 2% to 21% of the explained variance. In the subsequent models, the set of other health indicators also made a significant contribution, accounting for 3% to 13% of an additional variance. These findings are in support of Jylhä’s cognitive process model [17], in that subjective health evaluations were influenced by the indicators of other domains of health. Although several studies have explored the role of non-primary health indicators in subjective ratings of health, the scope of their assessments has been limited to the role of depressive symptoms in ratings of physical health [44]. Expanding the scope of investigation, the present study demonstrates the importance of considering a wider range of health indicators—both domain-specific and other indicators of physical, mental, oral, and cognitive health—to obtain a better understanding of the processes of subjective health assessment.

In all measures of self-ratings, the common finding was that domain-specific health indicators played a primary role and other health indicators played a secondary role. However, in the predictive model of self-rated cognitive health, the predictive power of the other health indicators exceeded that of the domain-specific health indicator. Although the MMSE score was a significant predictor of SRCH, it accounted for only 2% of the variance, whereas the amount of variance explained by other health indicators was over 13%. It was worthy to note that mental distress (*β* = −.25, *p* < .001) emerged as the most powerful driving factor of SRCH. Although this finding is in line with other studies [46,47], it calls for further investigation of the interplay between the constructs of mental and cognitive health.

In support of the cognitive process model [17] and the notion of social determinants of health [13,26], sociocultural factors made a substantial contribution to explaining self-assessment of health. In particular, acculturation and social network were significantly associated with health indicators (chronic disease, functional disability, problems with teeth or gums, and cognitive function) and self-ratings of all four domains of health. Individuals with higher levels of acculturation and larger networks with family and friends were not only in good health conditions but also had positive perceptions of health even when those health indicators were controlled. This pattern was consistent across all domains of health. The overall findings are in line with the literature highlighting the benefits of greater levels of acculturation and larger social networks to older immigrants [27–29] and suggest prioritizing those who are linguistically and socially isolated in the effort for health management and promotion.

Some limitations to this study should be noted. The use of a cross-sectional design and non-probability sampling strategies suggests caution in generalizing results to the national level and in drawing causal inferences. Despite efforts to recruit diverse group of older Korean Americans representing a wide range of health and socioeconomic status, randomized sampling was not possible due to difficulties in identifying the population. Thus, the findings are only suggestive and await further investigation. In addition, more complete assessments of health conditions (e.g., physical, psychiatric, oral, and neurological examination, diagnoses and testing by trained health professionals) would provide a stronger foundation for the validation of the self-reported measures. Furthermore, consideration should be given to broader levels of environmental variables such as neighborhood ethnic density and area health service availability.

Despite these limitations, however, the findings of the study contributed to our understanding of the multi-faceted nature of health and the processes of self-evaluation. Confirming the inter-connectedness of health domains and the roles of social determinants, the study highlights the importance of holistic health assessment that considers a wide range of health domains as well as sociocultural contexts. As self-perceptions of health are linked with health behaviors, chronic disease management, health service use, and mortality [14,15], poor ratings of health among those with limited resources deserve further attention.

## Figures and Tables

**Table 1:** Descriptive Characteristics of the Sample (*N* = 2,061)

	%	*M±SD*	Minimum-Maximum
**Demographic variables**			
Age		73.2±7.93	60–100
Gender			
Male	33.2		
Female	66.8		
Marital status			
Not married	39.2		
Married	60.8		
Education			
≤High school graduation	60.3		
>High school graduation	39 .7		
**Health indicators**			
Chronic disease		1.57±1.40	0–10
Functional disability		1.67±3.42	0–32
Mental distress (K6)		3.88±4.05	0–24
Problem with teeth or gums	21.0		
Cognitive function (MMSE)		26.7±2.91	10–30
**Sociocultural factors**			
Acculturation		12.2±7.06	0–35
Social network		15.5±6.05	0–30
Community cohesion		11.4±4.11	0–20
**Self-rated health assessment**^1^			
Self-rated physical health (SRPH)		3.02±1.24	1–5
Self-rated mental health (SRMH)		3.54±1.13	1–5
Self-rated oral health (SROH)		2.61±1.29	1–5
Self-rated cognitive health (SRCH)		3.15±1.13	1–5

1 Response format: poor (1), fair (2), good, (3), very good (4), excellent (5).

**Table 2:** Correlations among Study Variables

	1	2	3	4	5	6	7	8	9	10	11	12	13	14	15	16
1. Age	–															
2. Female	_−._12***	–														
3. Married	_−_.23***	−.26***	–													
4. >High school graduation	−.08**	−.29***	.16***	–												
5. Chronic disease	.27***	.11***	_−._16***	_−._17***	–											
6. Functional disability	.33***	.11***	_−._19***	_−._16***	.32***	–										
7. Mental distress	−.01	.10***	_−._12***	−.04	.16***	.19***	–									
8. Problem with teeth/gums	.18***	−.01	_−_.14***	−.14***	.25***	.21***	.21***	–								
9. Cognitive function	−.37***	_−._13***	.24***	.29***	−.21***	−.29***	−.07**	−.19***	–							
10. Acculturation	−.21***	−.10***	.21***	.36***	−.24***	−.26***	−.12***	−.22***	.30***	–						
11. Social network	_−._11***	.03	.20***	.13***	−.10***	−.13***	−.23***	_−._18***	.21***	.26***	–					
12. Community cohesion	.13***	.05*	−.01	−.03	.00	.03	_−._18***	.01	−.10***	−.02	.13***	–				
13. Self-rated physical health	−.23***	−.16***	.23***	.27***	−.47***	−.37***	−.27***	−.27***	.22***	.37***	.21***	.03	–			
14. Self-rated mental health	−.22***	_−_.11***	.20***	.27***	−.31***	−.32***	−.36***	−.26***	.29***	.36***	.24***	.06**	.62***	–		
15. Self-rated oral health	_−._17***	.02	.16***	.21***	−.23***	_−._18***	−.20***	_−._41***	.18***	.35***	.22***	.07**	.46***	.45***	–	
16. Self-rated cognitive health	_−._16***	−.10***	.15***	.26***	−.24***	−.23***	−.31***	−.26***	.25***	.37***	.24***	.07**	.51***	.67***	.51***	–

* *p* < .05.

** *p* < .01.

*** *p* < .001.

**Table 3:** Regression Models of Self-rated Physical, Mental, Oral, and Cognitive Health

	Standardized Regression Coefficient (β)															
	Self-rated Physical Health (SRPH)				Self-rated Mental Health (SRMH)				Self-rated Oral Health (SROH)				Self-rated Cognitive Health (SRCH)			
**Demographic variables**																
Age	−.19***	−.00	−.02	−.02	−.18***	−.19***	−.05*	−.05*	−.13***	−.07**	−.03	−.04	−.13***	−.08**	−.02	−.02
Female	−.09***	−.03	−.03	−.04	−.04	−.01	.02	.01	.09***	.07**	.10***	.08***	−.03	−.02	−.01	−.01
Married	.11***	.07***	.05*	.03	.10***	.07**	.04	.02	.11***	.08***	.06**	.03	.06**	.05	.00	.02
> High school	.22***	.15***	.14***	.10***	.23***	.23***	.16***	.12***	.22***	.17***	.16***	.10***	.24***	.20***	.17***	.11***
**Health indicators**																
Chronic disease		−.36***	−.33***	−.32***		—	−.13***	−.12***		—	−.10***	−.08***		—	−.09***	−.07**
Functional disability		−.25***	−.21***	−.19***		—	−.16***	−.14***		—	−.05*	−.02		—	−.12***	−.08***
Mental distress		—	−.16***	−.15***		−.35***	−.29***	−.27***		—	−.11***	−.08***		—	−.25***	−.23***
Teeth/gums problem		—	−.08***	−.06**		—	−.07**	−.05*		−.37***	−.32***	−.30***		—	−.10***	−.08***
Cognitive function		—	.01	.01		—	.10***	.08***		—	.03	.00		.15***	.11***	.07**
**Sociocultural factors**																
Acculturation				.14***				.14***				.20***				.20***
Social network				.05*				.04*				.06**				.07**
Community cohesion				.02				.02				.06**				.03
**Summary statistics**																
Changes in R^2^	.13***	.21***	.03***	.02***	.12***	.12***	.07***	.02***	.09***	.13***	.03***	.04***	.09***	.02***	.13***	.04***
Overall R^2^	.13***	.34***	.37***	.39***	.12***	.24***	.31***	.33***	.09***	.22***	.25***	.29***	.09***	.11***	.24***	.28***

* *p* < .05.

** *p* < .01.

*** *p* < .001.

## References

[1] Colby SL, Ortman JM: Projections of the Size and Composition of the US population: 2014 to 2060. Washington, DC: U.S. Census Bureau; 2015

[2] Jang Y, Yoon H, Park NS, Chiriboga DA: Health vulnerability of immigrants with limited English proficiency: A study of older Korean Americans. J Am Geriatr Soc. 2016;64(7): 1498–1502 27305524 10.1111/jgs.14199 PMC5441559

[3] Lum TY, Vanderaa JP: Health disparities among immigrant and non-immigrant elders: The association of acculturation and education. J Immigr Minor Health. 2010;12(5): 743–753 19184599 10.1007/s10903-008-9225-4

[4] U.S. Census Bureau: Selected Characteristics of the Native and Foreign-born Populations: 2012–2016 American Community Survey 5-year Estimates. Washington, DC: U.S. Census Bureau; 2016

[5] Đoàn LN, Takata Y, Sakuma KL, Irvin VL: Trends in clinical research including Asian American, Native Hawaiian, and Pacific Islander participants funded by the US National Institutes of Health, 1992 to 2018. JAMA Netw Open. 2019;2(7): e197432 31339543 10.1001/jamanetworkopen.2019.7432 PMC6659145

[6] Ghosh C: A national health agenda for Asian Americans and Pacific Islanders. JAMA. 2010;304(12): 1381–1382 20858884 10.1001/jama.2010.1358

[7] Islam NS, Khan S, Kwon S, Jang D, Ro M, Trinh-Shevrin C: Methodological issues in the collection, analysis, and reporting of granular data in Asian American populations: Historical challenges and potential solutions. J Health Care Poor Underserved. 2010;21(4): 1354–1381 21099084 10.1353/hpu.2010.0939 PMC3086449

[8] Jang Y, Choi EY, Rhee MK, Park NS, Chiriboga DA, Kim MT: Determinants of self-rated cognitive health among older Korean Americans. Gerontologist. 2020;60(2): 250–258 31618436 10.1093/geront/gnz134 PMC7317609

[9] Pandya C, McHugh M, Batalova J: Limited English Proficient Individuals in the United States: Number, Share, Growth, and Linguistic Diversity. Washington, DC: Migration Policy Institute; 2011

[10] López G, Ruiz NG, Patten E: Key Facts about Asian Americans, A diverse and Growing Population. Washington, DC: Pew Research Center; 2017

[11] Kim MT, Han HR, Shin HS, Kim KB, Lee HB: Factors associated with depression experience of immigrant populations: A study of Korean immigrants. Arch of Psychiatr Nurs. 2005;19(5): 217–225 16226673 10.1016/j.apnu.2005.07.004

[12] Eberst RM: Defining health: A multidimensional model. J of School Health. 1984;54(3): 99–104 6562286 10.1111/j.1746-1561.1984.tb08780.x

[13] Solar O, Irwin A: A conceptual framework for action on the social determinants of health. Geneva, Switzerland: World Health Organization; 2010

[14] DeSalvo KB, Bloser N, Reynolds K, He J, Muntner P: Mortality prediction with a single general self-rated health question: A meta-analysis. J of Gen Intern Med. 2006;21(3): 267–275 16336622 10.1111/j.1525-1497.2005.00291.x PMC1828094

[15] Ferraro KF, Kelley-Moore JA: Self-rated health and mortality among black and white adults: examining the dynamic evaluation thesis. J Gerontol B Psychol Sci Soc Sci. 2001;56(4): S195–205 11445612 10.1093/geronb/56.4.s195

[16] Idler E, Cartwright K: What do we rate when we rate our health? Decomposing age-related contributions to self-rated health. J Health Soci Behav. 2018;59(1): 74–93 10.1177/0022146517750137 29320638

[17] Jylhä M: What is self-rated health and why does it predict mortality? Towards a unified conceptual model. Soc Sci Med. 2009;69(3): 307–316 19520474 10.1016/j.socscimed.2009.05.013

[18] Ware JE Jr, Sherbourne CD: The MOS 36-item short-form health survey (SF-36): I. Conceptual framework and item selection. Med Care. 1992;30(6): 473–483 1593914

[19] Fleishman JA, Zuvekas SH: Global self-rated mental health: associations with other mental health measures and with role functioning. Med Care. 2007;45(7): 602–609 17571008 10.1097/MLR.0b013e31803bb4b0

[20] Jang Y, Yoon H, Chiriboga DA, Molinari V, Powers DA: Bridging the gap between common mental disorders and service use: The role of self-rated mental health among African Americans. Am J Geriatr Psychiatry. 2015;23(7): 658–665 24698444 10.1016/j.jagp.2014.02.010

[21] McAlpine DD, McCreedy E, Alang S: The meaning and predictive value of self-rated mental health among persons with a mental health problem. J Health Soc Behav. 2018;59(2): 200–214 29406825 10.1177/0022146518755485

[22] Shelley D, Russell S, Parikh NS, Fahs M: Ethnic disparities in self-reported oral health status and access to care among older adults in NYC. J Urban Health. 2011;88(4): 651–662 21850607 10.1007/s11524-011-9555-8 PMC3157507

[23] Wu B, Plassman BL, Liang J, Remle RC, Bai L, Crout RJ: Differences in self-reported oral health among community-dwelling black, Hispanic, and white elders. J Aging Health. 2011;23(2): 267–288 20858912 10.1177/0898264310382135 PMC3129602

[24] Cutler SJ: Worries about getting Alzheimer’s: Who’s concerned?. Am J Alzheimers Dis Other Demen. 2015;30(6): 591–598. 25657292 10.1177/1533317514568889 PMC10852650

[25] Knäuper B, Turner PA: Measuring health: improving the validity of health assessments. Qual Life Res. 2003;12(1): 81–89 12803314 10.1023/a:1023589907955

[26] Marmot M, Wilkinson R: Social determinants of health. 2^nd^ ed. Oxford: Oxford University Press; 2006

[27] Jang Y, Kim G, Chiriboga D, King-Kallimanis B: A bidimensional model of acculturation for Korean American older adults. J Aging Stud. 2007;21(3): 267–275 18670580 10.1016/j.jaging.2006.10.004 PMC2034290

[28] Park NS, Jang Y, Lee BS, Haley WE, Chiriboga DA: The mediating role of loneliness in the relation between social engagement and depressive symptoms among older Korean Americans: do men and women differ?. J Gerontol B Psychol Sci Soc Sci. 2013;68(2): 193–201 22929386 10.1093/geronb/gbs062 PMC3693603

[29] Zhang W, Ta VM: Social connections, immigration-related factors, and self-rated physical and mental health among Asian Americans. Soc Sci Med. 2009;68(12): 2104–2112 19427087 10.1016/j.socscimed.2009.04.012

[30] Chau S, Lai DW: The size of an ethno-cultural community as a social determinant of health for Chinese seniors. J Immigr Minor Health. 2011;13(6): 1090–1098 20686850 10.1007/s10903-010-9374-0

[31] Kawachi I, Subramanian SV, Kim D: Social capital and health. New York, NY: Springer; 2008

[32] Fillenbaum GG: OARS Multidimensional Functional Assessment Questionnaire and Services Supplement. Multidimensional Functional Assessment of older adults: The Duke Older American Resources and Services Procedures. Hillsdale NJ: Lawrence Erlbaum Associates; 1998

[33] Kessler RC, Andrews G, Colpe LJ, Hiripi E, Mroczek DK, Normand SL, : Short screening scales to monitor population prevalences and trends in non-specific psychological distress. Psychol Med. 2002;32(6): 959–976 12214795 10.1017/s0033291702006074

[34] Kessler RC, Barker PR, Colpe LJ, Epstein JF, Gfroerer JC, Hiripi E, : Screening for serious mental illness in the general population. Arch Gen Psychiatry. 2003;60(2): 184–189 12578436 10.1001/archpsyc.60.2.184

[35] Min SW, Lee SH: Validation of the K6/K10 scales of psychological distress and their optimal cutoff scores for older Koreans. Int J Aging Hum Dev. 2015;80(3): 264–282 26195502 10.1177/0091415015590316

[36] Folstein MF, Folstein SE, McHugh PR: “Mini-mental state”: a practical method for grading the cognitive state of patients for the clinician. J Psychiatr Res. 1975;12(3): 189–198 1202204 10.1016/0022-3956(75)90026-6

[37] Han JW, Kim TH, Jhoo JH, Park JH, Kim JL, Ryu SH, : A normative study of the Mini-Mental State Examination for Dementia Screening (MMSE-DS) and its short form (SMMSE-DS) in the Korean elderly. J Korean Geriatr Psychiatry. 2010;14(1): 27–37

[38] Lubben J, Blozik E, Gillmann G, Iliffe S, von Renteln Kruse W, Beck JC : Performance of an abbreviated version of the Lubben Social Network Scale among three European community-dwelling older adult populations. Gerontologist. 2006;46(4): 503–513 16921004 10.1093/geront/46.4.503

[39] Lubben J, Gironda M: Centrality of social ties to the health and well-being of older adults. In: Berkman B, Harooytoan LK, editors. Social work and health care in an aging world. New York, NY: Springer; 2003

[40] Hong M, Casado BL, Harrington D: Validation of Korean versions of the Lubben social network scales in Korean Americans. Clin Gerontol. 2011;34(4): 319–334

[41] Cagney KA, Glass TA, Skarupski KA, Barnes LL, Schwartz BS, Mendes de Leon CF: Neighborhood-level cohesion and disorder: measurement and validation in two older adult urban populations. J Gerontol B. 2009;64(3): 415–424 10.1093/geronb/gbn041 PMC2670251 19255089

[42] Bennett IM, Chen J, Soroui JS, White S: The contribution of health literacy to disparities in self-rated health status and preventive health behaviors in older adults. Ann Fam Med. 2009;7(3): 204–211 19433837 10.1370/afm.940 PMC2682976

[43] Coyle CE, Dugan E: Social isolation, loneliness and health among older adults. J Aging Health. 2012;24(8): 1346–1363 23006425 10.1177/0898264312460275

[44] Levinson D, Kaplan G: What does self rated mental health represent. J Public Health Res. 2014;3(3): 287 25553310 10.4081/jphr.2014.287 PMC4274494

[45] Alexopoulos GS, Kiosses DN, Heo M, Murphy CF, Shanmugham B, Gunning-Dixon F: Executive dysfunction and the course of geriatric depression. Biol Psychiatry. 2005;58(3): 204–210 16018984 10.1016/j.biopsych.2005.04.024

[46] Schweizer S, Kievit RA, Emery T, Henson RN: Symptoms of depression in a large healthy population cohort are related to subjective memory complaints and memory performance in negative contexts. Psychol Med. 2018;48(1): 104–114 28625188 10.1017/S0033291717001519 PMC5729845

[47] Yates JA, Clare L, Woods RT, MRC CFAS: Subjective memory complaints, mood and MCI: a follow-up study. Aging Ment Health. 2017;21(3): 313–321 26329364 10.1080/13607863.2015.1081150 PMC5351791

